# Supplementary material for: Causal Relationship between Aging and Anorexia Nervosa: A White-Matter-Microstructure-Mediated Mendelian Randomization Analysis
Source: Biomedicines. 2024 Aug 16;12(8):1874. doi: 10.3390/biomedicines12081874 (PMC11351342; doi:10.3390/biomedicines12081874)
Supplement: Supplementary file 1 [file biomedicines-12-01874-s001.zip › Supplementary Figure/Figure S8/Leaveoneout plot of MR analysis for TL to ubm-b-1546.pdf]

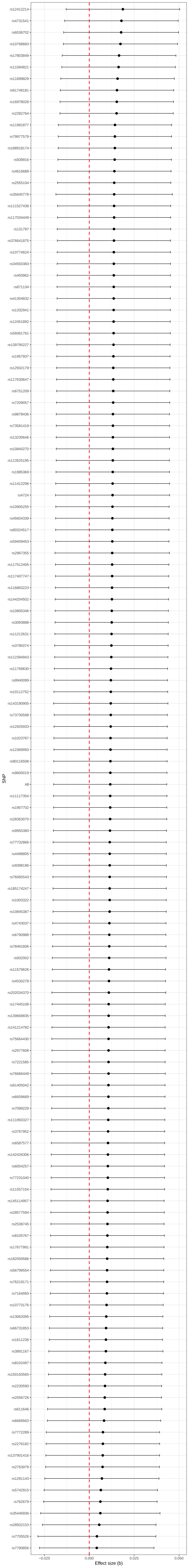

rs12412214
rs4731541
rs6536702
rs10768683
rs17803849
rs11584821
rs11699829
rs61748181
rs16978028
rs2282764
rs11991877
rs79977579
rs188918174
rs939916
rs4616688
rs2555104
rs35640778
rs111527438
rs117034449
rs131797
rs376641875
rs10774624
rs34550383
rs450962
rs871134
rs41304832
rs1332941
rs12451892
rs56061761
rs139795227
rs1957937
rs12932179
rs117630647
rs6751209
rs7209057
rs9878436
rs73581419
rs13230646
rs10840270
rs113525195
rs1985369
rs11412296
rs4724
rs10905255
rs45604339
rs80324517
rs59409453
rs2967355
rs117512405
rs117407747
rs116863223
rs144204502
rs10805346
rs3093888
rs11212631
rs3785074
rs112394943
rs11769630
rs9940099
rs10112752
rs143190905
rs73730598
rs12925933
rs1023767
rs12369950
rs80116508
rs9600019
All
rs11117354
rs1907702
rs28363070
rs9955360
rs77732866
rs4498805
rs9398196
rs76065543
rs185174247
rs1003322
rs10845387
rs4743037
rs6790988
rs78491606
rs932002
rs11579626
rs4530278
rs202034370
rs17445108
rs139669835
rs141214782
rs75664430
rs2977608
rs7221585
rs76666449
rs61405042
rs6659669
rs7099229
rs111950327
rs3767952
rs6587577
rs142426306
rs6054257
rs77231040
rs11557154
rs145114957
rs28577594
rs2538745
rs8105767
rs17677991
rs182059586
rs56799554
rs76219171
rs7164950
rs10773176
rs13062095
rs66731853
rs1611236
rs3891167
rs8102497
rs150150565
rs2230590
rs2056726
rs611646
rs6669563
rs7772289
rs2276182
rs137901416
rs2763979
rs1291143
rs5742915
rs762679
rs35446936
rs28502153
rs7705526
rs7790856
SNP
-0.025
0.000
0.025
0.050
Effect size (b)
